# Simultaneous realization of slow and fast acoustic waves using a fractal structure of Koch curve

**DOI:** 10.1038/s41598-018-19797-x

**Published:** 2018-01-24

**Authors:** Jin Ding, Li Fan, Shu-yi Zhang, Hui Zhang, Wei-wei Yu

**Affiliations:** Lab of Modern Acoustics, Institute of Acoustics, Nanjing University, Nanjing, 210093 P. R. China

## Abstract

An acoustic metamaterial based on a fractal structure, the Koch curve, is designed to simultaneously realize slow and fast acoustic waves. Owing to the multiple transmitting paths in the structure resembling the Koch curve, the acoustic waves travelling along different paths interfere with each other. Therefore, slow waves are created on the basis of the resonance of a Koch-curve-shaped loop, and meanwhile, fast waves even with negative group velocities are obtained due to the destructive interference of two acoustic waves with opposite phases. Thus, the transmission of acoustic wave can be freely manipulated with the Koch-curve shaped structure.

## Introduction

The manipulation of wave transmission has attracted considerable attention in the fields of electromagnetics, optics and acoustics, in which numerous endeavors were provided to achieve extremely fast or slow waves. Superluminal transmissions and negative group velocities of light were obtained in highly nonlinear fibers and gaseous nanolayers^1,2^. On the contrary, slow light was produced in a dendritic cell cluster metasurface waveguide and dual coupled-resonator system^3,4^. Encouraged by the achievements in the control of electromagnetic waves and light, variant methods were adopted to manipulate the transmissions of acoustic waves. First, faster-than-light acoustic waves were created in a Herschel–Quincke tube, in which the envelope of the output acoustic pulse preceded that of the input pulse^5^. Then, extremely large and even negative group velocities were also realized in a suspension of elastic microspheres^6^. On the other hand, studies were performed for the realizations of slow waves and energy trapping. An acoustic metamaterial composed of an array of grooves perforated on a rigid bar was presented to trap acoustic waves at separate locations according to frequencies, which was called to be a phenomena of rainbow trapping^7^. Defected phononic crystals were widely used to produce slow acoustic waves. By introducing a line defect in a triangular array of aluminum cylinders, slow waves were obtained at audio frequencies^8^. Furthermore, on the analogy of dual coupled resonators used to produce slow light^4^, an acoustic metamaterial was created based on a series of detuned Helmholtz resonator pairs arranged along a tube. When the resonant frequencies of the resonator pairs are close to each other, slow sound waves were achieved at a frequency between both resonant frequencies^9,10^, which resembles the phenomenon of electromagnetically induced transparency in optics^11^. Furthermore, a simple and compact structure of one string of side pipes arranged along a waveguide was present to obtain diverse group velocities, as slow acoustic waves, fast acoustic wave and even negative group velocity^12^. Additionally, the transmissions of acoustic waves in solids^13,14^ and water^15^ were also manipulated to change the relative refractive indices and acoustic velocities, in which distinct acoustic lens were created. Then, by controlling the velocities of acoustic waves traveling in different directions, anisotropic swirling surface acoustic waves were produced and acoustic vortices were realized in solids^16^. Currently, the structures with labyrinthic or helical paths were used to slow down the propagations of acoustic waves on the basis of phase shifts originating from the transmissions along the curved and lengthened paths^17–22^, which exhibited the potentials in sound blocking^18,19^, directional sensing^20^, broadband attenuation^21^, topological insulation^22^. Similar to labyrinthic structures, fractal structures, as the Koch curve^23,24^, Sierpinski fractal^25,26^, and Hilbert curve^27,28^ were also used to manipulate the transmissions of waves, which have been widely applied in electromagnetic metamaterials to obtain high impedance surfaces, broadband polarization insensitive absorption, harmonic waves suppression, directivity enhancement, and etc.

In this work, we create an acoustic metamaterial with the structure resembling a traditional fractal structure, the Koch curve, in which multiple transmission paths with different lengths are provided. Thus, on the basis of distinct mechanisms induced by the acoustic transmissions along different paths, diverse group velocities of the acoustic fields are obtained in the metamaterial, which exhibits potential applications in the fields requiring the manipulation of group velocities of acoustic waves, as acoustic filters, macrosonics application, precise spatial-spectral control, perfect absorbing, nonlinear enhancement and so on.

## Results

**Figure 1:**
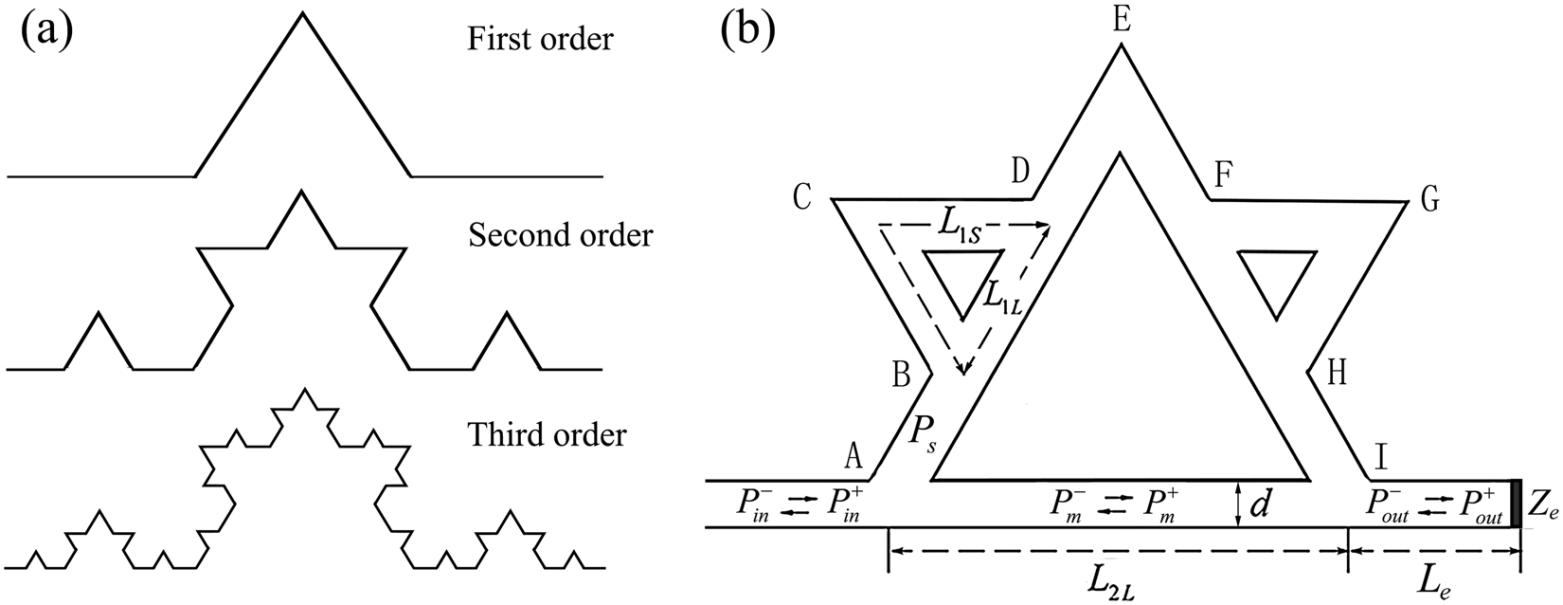
Sketch of Koch curve and QKCM. (**a**) The first, second and third order Koch curve. (**b**) The sketch of the designed QKCM, in which the structural parameters are *L*_AI_ = 116 cm, *L*_AB_ = 43 5 cm, *L*_BD_ = 35.5 cm, *L*_DE_ = 39 cm, *L*_EF_ = 39.5 cm, *L*_FH_ = 35.5 cm, *L*_HI_ = 43.5 cm, *L*_BD_ = 36.5 cm, *L*_CD_ = 36.5 cm, *L*_FG_ = 36.5 cm, *L*_GH_ = 36.5 cm, *d* = 3 cm.

Figure 1a shows the sketch of the standard Koch curves of the first three orders, which exhibit a characteristic of self-similarity. Figure 1b indicates an acoustic metamaterial created based on the second order Koch curve, in which the removed bases (AI, BD and FH) of the triangles in the standard Koch curve are recovered. Therefore, the structure is defined to be a quasi-Koch-curve shaped metamaterial (QKCM). The acoustic waves bifurcate and converge repeatedly while transmitting in the QKCM, exhibiting complex propagations along different paths.

**Figure 2:**
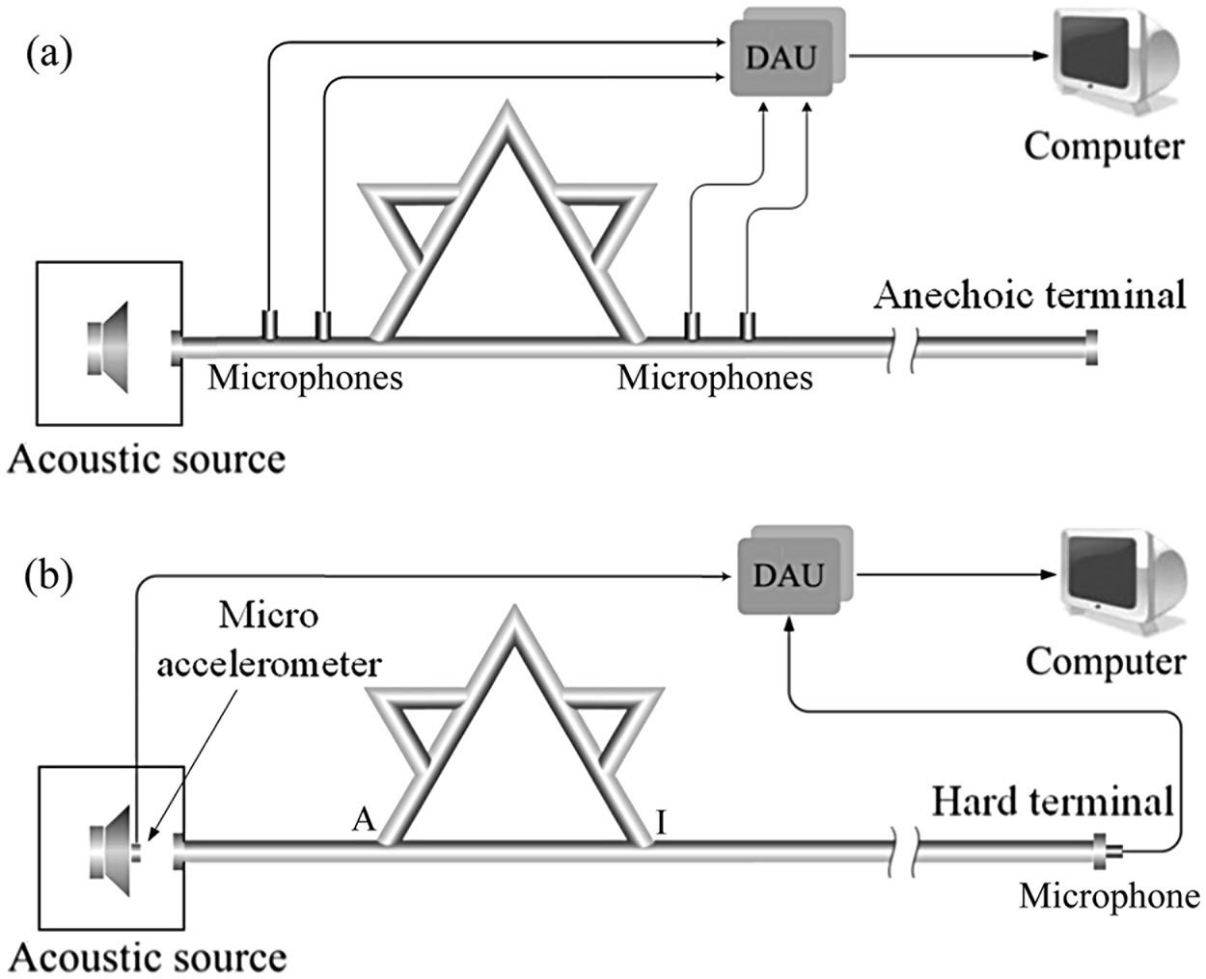
Experimental apparatus. (**a**) The experimental apparatus to measure the transmission of the QKCM with four-microphone method. (**b**) The experimental system to measure the group velocity in the QKCM. The structural parameters of the QKCMs in both systems are the same as those shown in Fig. 1.

**Figure 3:**
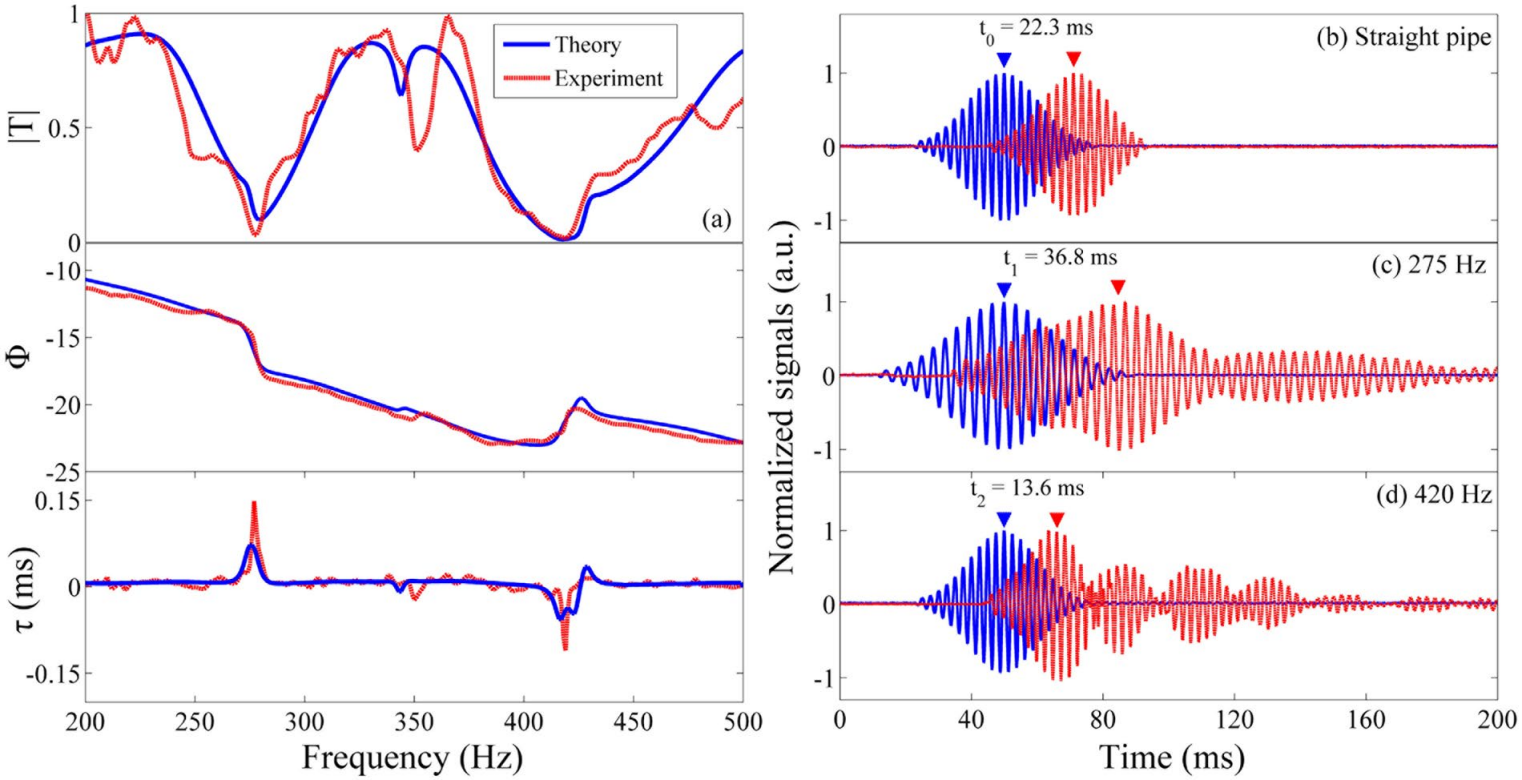
Theoretical and experimental results. (**a**) The theoretical (solid line) and experimental (dashed line) transmission amplitudes, phases and phase times. The theoretical and experimental results are both obtained on the basis of the parameters in the QKCM shown in Fig. 1b. (**b**) Measured acoustic pulses in a straight waveguide with the same length as the QKCM as a gauge to evaluate the group velocity. (**c**) and (**d**) Measured signals in the QKCM using the exciting of Gaussian pulses with the central frequencies of 275 Hz and 420 Hz, respectively. The solid line (blue) indicates the signal measured by the accelerator attached on the loudspeaker diaphragm and the dashed line (red) indicates the signal detected by the microphone set up at the hard terminal.

Two experimental systems are established to measure the performance of the QKCM. The apparatus in Fig. 2a is used to measure the transmission *T*_*r*_ of the QKCM, which is set up on the basis of four-microphone method^29^. Then, from the phase *ϕ* of the transmission coefficient *T*_*r*_, the phase time can be calculated by *τ* = −*∂ϕ/∂ω*. The measured transmissions and phase times for the QKCM are compared to those simulated with a theoretical model (see Methods), as shown in Fig. 3a. It can be observed that the theoretical and experimental results demonstrate two abrupt jumps in the phase *ϕ* of transmission, indicating strong dispersion, which result in a high peak of the phase time *τ* at 275 Hz and a deep valley at 420 Hz. The group velocity *v*_*g*_ of an acoustic pulse transmitting through the QKCM, which is independent of the phase velocity, can be expressed by^5^:

1 $${v}_{g}=({v}_{c}{\rm{\Delta }}L)/({\rm{\Delta }}L+{v}_{c}{\rm{\Delta }}t),$$

in which *v*_*c*_ is the speed of an acoustic pulse propagating through a straight waveguide, Δ*L* is the effective transmitting distance of the pulse, which must be identical in the straight waveguide and the QKCM. Δ*t* is the difference between the traveling times in the QKCM and the straight waveguide, which is related to the phase time. From Eq. (1), it can be observed that when Δ*L* + *v*_*c*_Δ*t* → 0, a large group velocity is obtained, and furthermore, when Δ*t* is sufficiently negative, resulting in Δ*L* + *v*_*c*_Δ*t* < 0, the group velocity *v*_*g*_ can be negative. Therefore, it is predicted that a negative group velocity can be achieved near the negative valley of the phase time at 420 Hz. On the other hand, Eq. (1) demonstrates that *v*_*g*_ decreases with the increase of Δ*t*, and thus, a slow wave can be obtained in the vicinity of the high peak of the phase time at 275 Hz.

To demonstrate the manipulation of group velocities with the QKCM, we set up an apparatus to measure the traveling time of an acoustic pulse in the QKCM, which is indicated in Fig. 2b. A sinusoidal signal with the frequency of *f*_0_ enveloped by a Gaussian pulse with the width of 21 sine cycles is adopted as an excitation, which is detected by a micro accelerometer established on the diaphragm of the loudspeaker. The output signal is measured with a microphone set up at the hard terminal and the distance between node I and the terminal must be sufficiently long, which is 5 m in our experiments, in this case, the echo waves can be separated from the detected signal.

The input and output Gaussian pulses detected by the accelerator and microphone are shown in Fig. 3b–d, from which the transmitting time from the loudspeaker to the hard terminal can be obtained. Since the acoustic waves in a straight waveguide are non-dispersive, the transmission of a Gauss pulse with the central frequency *f*_0_ = 420 Hz along a straight waveguide with the same length of the QKCM is measured as the criterion, which is shown in Fig. 3b. It can be observed that the output signal lags behind the input one, indicating a traveling time of *t*_0_ = 22.3 *ms*. Then, according to the transmission distance of *L* = 7.57 *m*, the group velocity is calculated to be 339 *m*/*s*, which is in accordance to the acoustic velocity in air.

Figure 3c indicates the transmission of an acoustic pulse with the central frequency of 275 Hz, which is the frequency for the high peak of the phase time measured in the QKCM. It is observed that the output signal lags far behind the input one, which indicates a transmitting time of *t*_1_ = 36.8 *ms*. Thus, the difference between the transmission times in the QKCM and the straight waveguide is calculated to be Δ*t* = *t*_1_ − *t*_0_ = 14.5 *ms*, which demonstrates that the pulse in the QKCM travels much slower than it propagates along the straight waveguide. According to the effective transmission path Δ*L* = 116 cm from A to I, the group velocity can be evaluated with Eq. (1), which is *v*_*g*_ = 64.7 *m*/*s*. The group velocity is extremely low and solely 1/5 of the acoustic velocity in the air, which demonstrates a slow wave. Additionally, it can be observed that a low peak of phase time appears at 429 Hz, which also exhibits a slow wave, while the group velocity *v*_*g*_ = 125.8 *m*/*s* is larger than that achieved at 275 Hz.

Furthermore, Fig. 3d shows the transmission of an acoustic pulse with *f*_0_ = 420 Hz, which is the frequency for the valley of the phase time. The input pulse takes *t*_2_ = 13.6 *ms* to reach the hard terminal, and the time difference is Δ*t* = *t*_2_ − *t*_0_ = −8.7 *ms*, which demonstrates that the pulse in the QKCM travels much faster than it propagates along the straight waveguide. According to Eq. (1), if Δ*L* + *v*_*c*_Δ*t* < 0, namely, Δ*t* < −3.5 *ms*, a negative group velocity *v*_*g*_ < 0 can be obtained. Thus, the time difference of −8.7 *ms* demonstrates a negative group velocity of *v*_*g*_ = −219.8 *m*/*s*. It must be noted that the negative group velocity does not violate causality indicating that the cause of an even precedes the effect because the transmission velocity of information carried by a wave packet is determined by the velocity of the front of the packet, which was defined to be Sommerfeld precursor^30^. As shown in Fig. 3d, the Sommerfeld precursor of the signal detected by the microphone lags that of the input signal by 21.2 *ms*, which obeys causality.

It must be noted that the manipulation of group velocity is realized on the basis of spectral rephrasing^30^. Due to the strong dispersion, the phase advances to different degrees at the frequencies near the peak and valley of the phase time, which results in the reshaping of the envelope of wave packet. Therefore, a distortion in the output wave packet is inevitable^16,30^. An expanding of the envelope of the output signal can be observed when a slow wave is obtained. Especially, when a negative group velocity is observed, the orders of the wave fronts and envelopes of the input and output signals are inversed, which results in distortion and tails of the output signal^30^.

## Discussion

**Figure 4:**
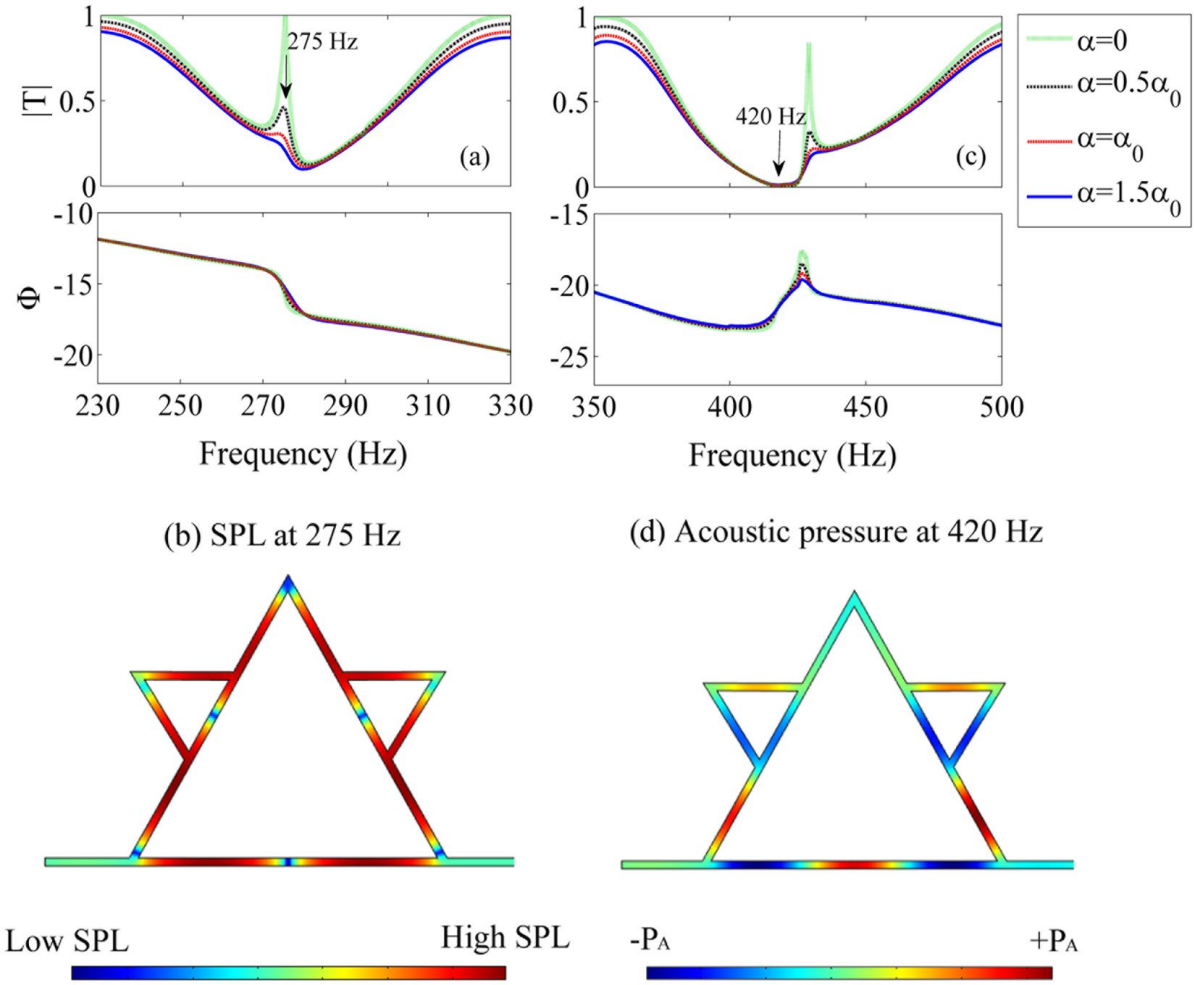
Transmissions and acoustic fields in the QKCM. (**a**) and (**c**) The theoretical transmissions calculated with Eq. (3) based on different attenuation coefficients in which ${\alpha }_{0}=2.78\times {10}^{-5}\sqrt{f}/0.5d$ and the distributions of (**b**) SPL at 275 Hz and (**d**) acoustic pressure at 420 Hz simulated with COMSOL software.

It is demonstrated that the single structure of the QKCM can simultaneously produces slow waves and fast waves, even with negative group velocities. The diversity of the group velocities is induced by the interaction of acoustic waves traveling along different paths in the QKCM, and thus, the slow and fast waves are obtained on the basis of distinct mechanisms. To study the mechanisms for the different group velocities in the QKCM, we calculate the transmissions based on different attenuation coefficients of the system, which are shown in Fig. 4a and c. It can be observed that the transmission obtained in the vicinity of 275 Hz, the frequency for the slow wave, is greatly influenced by the attenuation *α* in the QKCM. A transmission peak arises at 275 Hz when the attenuation coefficient is small, which can reach 1 in a lossless system with *α* = 0. The peak in the amplitude of transmission cannot be observed under a large attenuation coefficient, and thus, it can be deduced that the transmission peak is related to resonance effect. However, the jump in the transmission phase preserves under a large attenuation, and the slow wave can be observed in the experiments. Figure 4b indicates the distribution of sound pressure level (SPL) at 275 Hz in the metamaterial, which shows that the SPL in the Koch-curve-shaped loop between nodes A and I is considerably higher than that outside of the loop. Therefore, it can be known that the phase jump are induced by the resonance of the quasi-Koch-curve structure, which traps the acoustic energy in the complex loop between nodes A and I. Since resonance is influenced by attenuation, the resonance-induced transmission peak is considerably decreased by a large damping. Similarly, it is found that the small peak of the phase time obtained at 429 Hz is also induced by resonance because the transmission peak decreases with the increase of attenuation. On the other hand, Fig. 4c shows that the transmission valley in the vicinity of 420 Hz is marginally influenced by the attenuation, which indicates that the negative group velocity achieved at 420 Hz is independent of resonance. It can be found in Fig. 4d that the acoustic waves traveling along the side structure and the main tube converge with opposite phases at node I. Thus, the transmission valley and negative group velocity at 420 Hz is induced by the destructive interference of two acoustic waves with opposite phases. As a result, it is demonstrated that diverse group velocities, from extremely slow waves to fast waves even with negative group velocities, are produced by different mechanisms in the presented QKCM.

**Figure 5:**
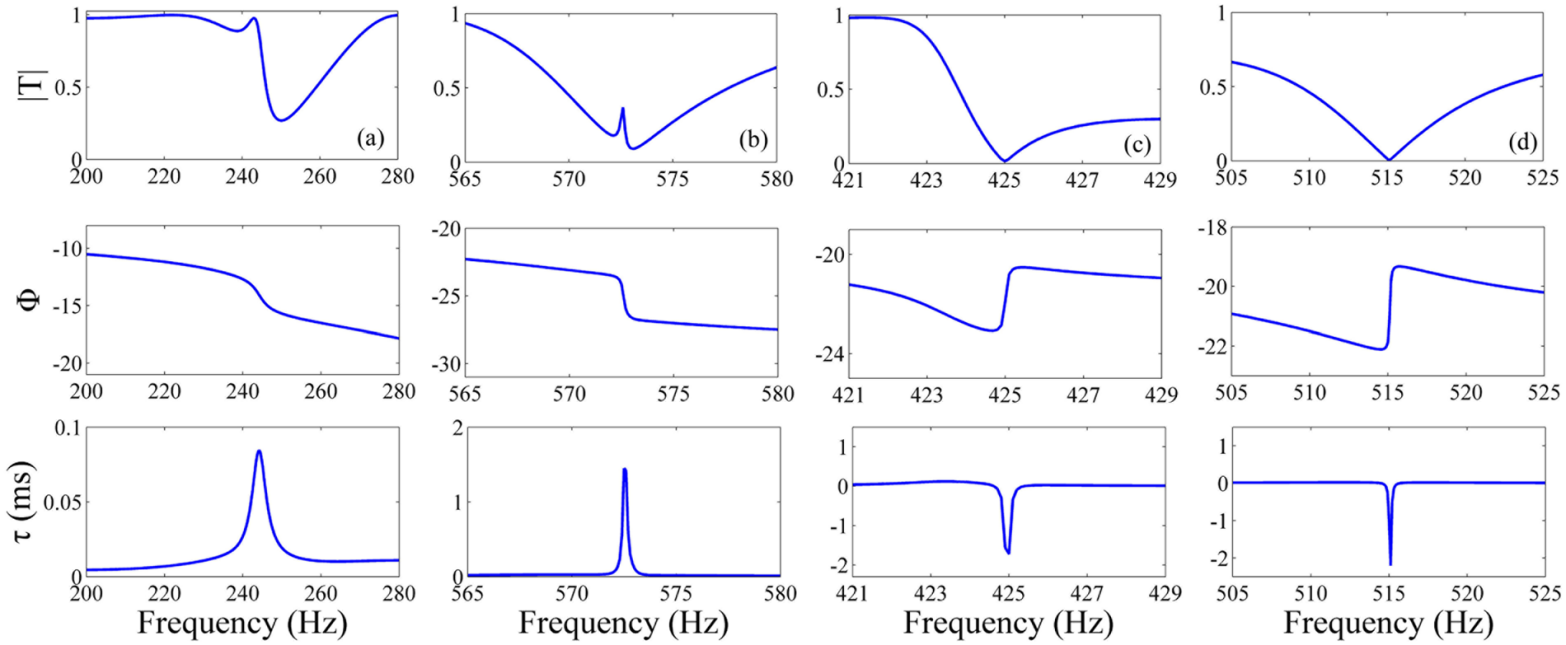
Theoretical transmissions and phase times in a three-ordered QKCM. Theoretical transmissions and phase times in the vicinities of (**a**) 243 Hz, (**b**) 573 Hz, (**c**) 425 Hz and (**d**) 515 Hz obtained in a metamaterial with the shape resembling a three-order Koch curve, in which the side length of the largest triangle is 120 cm.

Furthermore, by designing a metamaterial based on a high-order Koch curve, we can obtain slow and fast waves at more frequencies. Figure 5 exhibits the calculated transmission and phase time in a metamaterial created on the basis of a three-order Koch curve. Two peaks can be observed in the phase time at the frequencies of 243 Hz and 573 Hz, demonstrating slow waves. Resembling that in the QKCM based on the two-order Koch curve, at both frequencies, transmission peaks can be found, which exhibit that the slow waves are induced by resonance effect. On the other hand, two valleys of the phase time occur at the frequencies of 425 Hz and 515 Hz, which exhibit fast waves with negative group velocities, and the low transmissions at both frequencies demonstrate that the fast waves result from destructive interference of acoustic waves. It is shown that we can achieve slow and/or fast waves at distinct frequencies by using a QKCM on the basis of different orders of fractal structures, which realizes free manipulation of acoustic transmissions.

In summary, we create an acoustic metamaterial based on a fractal structure, the Koch curve, and establish a theoretical model to describe the acoustic transmission in the structure resembling an arbitrary order Koch curve. The QKCM can simultaneously produces slow acoustic waves and fast waves even with negative group velocities on the basis of different physical mechanisms. First, the resonance of the quasi-Koch-curve structure traps the acoustic energy in the complex loop and creates a slow wave. Additionally, due to the destructive interference originating from acoustic waves travelling along different paths, a negative group velocity is realized. Therefore, multiple transmission paths for acoustic waves are produced based on one simple structure, and thus, diverse group velocities are achieved owing to the interaction of acoustic waves transmitting along different paths, which can be applied in the fields requiring the manipulation of acoustic transmissions.

## Methods

### Theoretical model and numerical simulations

As indicated in the QKCM shown in Fig. 1b, by defining a transfer matrix **T**_**K**_, the relationship between the input (*P*_*in*_) and output (*P*_*out*_) acoustic waves in the QKCM can be simply expressed to be ${[{P}_{out}^{+},{P}_{out}^{-}]}^{T}={{\bf{T}}}_{{\bf{K}}}{[{P}_{in}^{+},{P}_{in}^{-}]}^{T}$. Then, at the input and output of the metamaterial, A and I, we have the continuities of acoustic pressures ${{p}_{in}|}_{A}={{p}_{m}|}_{A}={{p}_{s}|}_{A}$, ${{p}_{m}|}_{I}={{p}_{out}|}_{I}={{p}_{s}|}_{I}$ and of velocities ${{v}_{in}|}_{A}={{v}_{m}|}_{A}+{{v}_{s}|}_{A}$, ${{v}_{m}|}_{I}={{v}_{out}|}_{I}+{{v}_{s}|}_{I}$. Similarly, the acoustic transmissions in the branch structure from node A to E and from node E to I are also determined by the same continuity conditions of acoustic pressure and velocity, from which the transfer matrix for the small triangular loop (BCD or FGH) can be deduced to be:

2 $${{\bf{T}}}_{{\bf{L}}}={[\begin{array}{cc}-({\alpha }_{1L}+{\alpha }_{1S})+\frac{{(1+{\beta }_{1L}+{\beta }_{1S})}^{2}}{4({\alpha }_{1L}+{\alpha }_{1S})} & -({\alpha }_{1L}+{\alpha }_{1S})-\frac{1-{({\beta }_{1L}+{\beta }_{1S})}^{2}}{4({\alpha }_{1L}+{\alpha }_{1S})}\\ ({\alpha }_{1L}+{\alpha }_{1S})+\frac{1-{({\beta }_{1L}+{\beta }_{1S})}^{2}}{4({\alpha }_{1L}+{\alpha }_{1S})} & ({\alpha }_{1L}+{\alpha }_{1S})-\frac{{(1-{\beta }_{1L}-{\beta }_{1S})}^{2}}{4({\alpha }_{1L}+{\alpha }_{1S})}\end{array}]}^{-1},$$

in which ${\alpha }_{1S,L}=1/({e}^{jkl{L}_{1S,{\rm{L}}}}-{e}^{-jk{L}_{1S,L}})$ and ${\beta }_{1S,L}=(1+{e}^{-2jk{L}_{1S,L}})/(1-{e}^{-2jk{L}_{1S,L}})$, with *k* = *ω*/*c*_0_ + *jα*_0_ as the wave number including the attenuation *α*_0_ in the metamaterial, where *ω* and *c*_0_ are the angular frequency and acoustic velocity in air, respectively. Then, the transfer matrix **S** describing the acoustic transmitting along the side structure from node A to I can be obtained by successively multiplying the transfer matrices for two small triangular loops and four straight pipes. It can be proven that **S** is a diagonal complex conjugate matrix, ${S}_{11}={S}_{22}^{\ast }$ and ${S}_{12}={S}_{21}^{\ast }$, with the determinant |**S**| = 1. Then, applying the continuity conditions at nodes A and I, we obtain the transfer matrix **T**_**K**_ for the whole QKCM:

3 $$\begin{array}{c}{{\bf{T}}}_{{\bf{K}}}=[\begin{array}{c}-({\alpha }_{2L}+{\alpha }_{2S})+\frac{(1+{\beta }_{2L}+{\beta }_{2S})(1+{\beta }_{2L}+{\beta ^{\prime} }_{2S})}{4({\alpha }_{2L}+{\alpha }_{2S})}\\ ({\alpha }_{2L}+{\alpha }_{2S})+\frac{(1-{\beta }_{2L}-{\beta }_{2S})(1+{\beta }_{2L}+{\beta ^{\prime} }_{2S})}{4({\alpha }_{2L}+{\alpha }_{2S})}\end{array}\\ {\begin{array}{c}-({\alpha }_{2L}+{\alpha }_{2S})-\frac{(1+{\beta }_{2L}+{\beta }_{2S})(1-{\beta }_{2L}-{\beta ^{\prime} }_{2S})}{4({\alpha }_{2L}+{\alpha }_{2S})}\\ ({\alpha }_{2L}+{\alpha }_{2S})-\frac{(1-{\beta }_{2L}-{\beta }_{2S})(1-{\beta }_{2L}-{\beta ^{\prime} }_{2S})}{4({\alpha }_{2L}+{\alpha }_{2S})}\end{array}]}^{-1},\end{array}$$

in which, ${\alpha }_{2S}=1/2[{\rm{Im}}\,({S}_{12})-{\rm{Im}}\,({S}_{11})]$, ${\beta }_{2S}=[{\rm{Re}}({S}_{11})+{\rm{Re}}({S}_{12})]/[{\rm{Im}}({S}_{12})-{\rm{Im}}({S}_{11})]$, and ${\beta }_{2S}^{\prime} =[{\rm{Re}}({S}_{11})-{\rm{Re}}({S}_{12})]/[{\rm{Im}}({S}_{12})-{\rm{Im}}({S}_{11})]$, where Re() and Im() indicate the real and imaginary parts, respectively. Because the transfer matrix for the straight tube from A to I is ${\bf{T}}=[{T}_{11},{T}_{12};{T}_{21},{T}_{22}]=[{e}^{-jk{L}_{2L}},0;0,{e}^{jk{L}_{2L}}]$, *α*_2*L*_ and *β*_2*L*_ can be expressed with the same forms as *α*_2*S*_ and *β*_2*S*_ by replacing *S*_*ij*_ with *T*_*ij*_ (*i*, *j* = 1, 2). Additionally, Eq. (2) can also be rewritten with the same form as Eq. (3) by considering that *β*_1*S*_ = *β′*_1*S*_ in the small triangular loops. Therefore, we obtain a universal equation (3) to describe the acoustic transmission in a by-pass structure with multiple paths. Furthermore, according to the self-similarity of the Koch curve, we can achieve the transfer matrix in a structure with the shape resembling the Koch curve of an arbitrary order by successively using Eq. (3). Then, the transmission coefficient for the QKCM can be expressed to be ${T}_{r}={P}_{out}^{+}/{P}_{in}^{+}={({T}_{Ki11}+{r}_{p}{T}_{Ki12})}^{-1}$, in which *T*_K*i*11_ and *T*_*Ki*12_ are the components of the first row in the inverse matrix of **T**_**K**_, and *r*_*p*_ is the reflection coefficient at the terminal of the QKCM, which is determined by the load impedance *Z*_*e*_ and the distance *L*_*e*_.

### Experimental apparatus

As shown in Fig. 2a, a loudspeaker is used to input wide-band acoustic signals into the metamaterial and four mini microphones with the diameters of 6 mm (B&K 2670) are set up in pairs at the input and output of the QKCM with an anechoic termination. The signals gained from every microphone are collected by a data acquisition unit (DAU) and input into a computer. By means of four-microphone method, the forward and backward components in the input and output acoustic fields can be extracted from the measured signals, then the transmission of QKCM is achieved.

In the measurement of the group velocities in the QKCM, an apparatus is set up to measure the traveling time of an acoustic pulse, as indicated in Fig. 2b. A sinusoidal signal with the frequency of *f*_0_ enveloped by a Gaussian pulse with the width of 21 sine cycles is adopted as an excitation. To accurately determine the transmitting time of the Gaussian pulse, the influence of the echo on the detected signals must be eliminate. Therefore, a micro accelerometer is established on the diaphragm of the loudspeaker to detect the input pulse, which cannot sense the reflected acoustic waves from node A of the QKCM. The output signal is measured with a microphone set up at the hard terminal and the distance between node I and the terminal must be sufficiently long, which is 5 m in our experiments, in this case, the echo waves can be separated from the detected signal.
